# Effects of Vitamin D Supplementation on Body Composition and Metabolic Risk Factors in Men: A Randomized Controlled Trial

**DOI:** 10.3390/nu11081894

**Published:** 2019-08-14

**Authors:** Elisabeth Lerchbaum, Christian Trummer, Verena Theiler-Schwetz, Martina Kollmann, Monika Wölfler, Stefan Pilz, Barbara Obermayer-Pietsch

**Affiliations:** 1 Division of Endocrinology and Diabetology, Department of Internal Medicine, Medical University of Graz, Auenbruggerplatz 15, 8036 Graz, Austria; 2 Division of Obstetrics, Department of Obstetrics and Gynecology, Medical University of Graz, Auenbruggerplatz 14/1, 8036 Graz, Austria

**Keywords:** vitamin D, randomized controlled trial, insulin sensitivity, obesity, body composition

## Abstract

Vitamin D might play a role in metabolic processes and obesity. We therefore examined vitamin D effects on metabolic markers and obesity in a randomized controlled trial (RCT). This is a post-hoc analysis of the Graz Vitamin D&TT-RCT, a single-center, double-blind, randomized placebo-controlled trial. We included 200 healthy men with serum 25-hydroxyvitamin D (25(OH) D) levels <75 nmol/L. Subjects received 20,000 IU of vitamin D3/week (*n* = 100) or placebo (*n* = 100) for 12 weeks. Outcome measures were metabolic markers, anthropometric measures, and body composition assessed by Dual-energy X-ray absorptiometry. One-hundred and ninety-two men completed the study. We found a significant treatment effect on fasting glucose/fasting insulin ratio (−5.3 (−10.4 to −0.2), *p* = 0.040), whereas we observed no significant effect on the remaining outcome parameters. In subgroup analyses of men with baseline 25(OH)D levels <50 nmol/L (*n* = 80), we found a significant effect on waist circumference (1.6 (0.3 to 2.9) cm, *p* = 0.012), waist-to-hip ratio (0.019 (0.002 to 0.036), *p* = 0.031), total body fat (0.029 (0.004 to 0.055) %, *p* = 0.026), and android fat (1.18 (0.11 to 2.26) %, *p* = 0.010). In middle-aged healthy men, vitamin D treatment had a negative effect on insulin sensitivity. In vitamin D deficient men, vitamin D has an unfavorable effect on central obesity and body composition.

## 1. Introduction

Vitamin D is well known for its effects on calcium and bone metabolism [1]. In addition, there is evidence from observational studies showing an association of a poor vitamin D status with various conditions including decreased fertility [2], hypogonadism [3], obesity, metabolic disorders including insulin resistance and type 2 diabetes mellitus [4,5], and cardiovascular disease [6]. These cross-sectional associations with cardiovascular risk factors might explain why vitamin D deficient individuals are at increased risk of mortality [7,8]. Underlying mechanisms might be beneficial effects of vitamin D on oxidative stress and inflammation as well as vitamin D related epigenetic alterations associated with insulin resistance and type 2 diabetes mellitus [9]. Therefore, vitamin D deficiency might accelerate the formation of insulin resistance [9].

Despite promising evidence from observational studies, evidence from randomized controlled trials (RCTs) regarding vitamin D effects on cardiovascular risk factors such as obesity, insulin sensitivity, or serum lipids is inconsistent [10,11,12,13]. Whether vitamin D has beneficial or harmful effect or possibly both is currently unclear. In detail, previous RCTs revealed no positive vitamin D effect on obesity or body composition [13], and inconsistent results regarding vitamin D effects on insulin resistance [10,14,15]. Recently, we found an unfavorable effect of vitamin D supplementation on insulin sensitivity assessed by quantitative insulin sensitivity check index (QUICKI) in 100 healthy middle-aged men with 25-hydroxyvitamin D (25(OH)D) levels <75 nmol/L at baseline participating in the Graz Vitamin D&TT-RCT [10], whereas no significant effect was found in 100 men with low total testosterone (TT) levels at baseline [15]. In contrast, a recent meta-analysis found a significant positive effect on insulin resistance among subjects with type 2 diabetes mellitus [16].

Given previous inconsistent results of vitamin D supplementation on metabolic markers, we performed a post-hoc analysis of the Graz Vitamin D&TT-RCT in order to (1) re-evaluate our previous inconsistent results regarding insulin sensitivity in a larger cohort including men with high as well as with low TT levels at baseline (*n* = 200) and (2) to analyze vitamin D effects on obesity assessed by anthropometric measurements and Dual-energy X-ray absorptiometry (DXA) in this cohort, as changes in body composition might mediate vitamin D effects on metabolic parameters.

## 2. Materials and Methods

This study is a post-hoc analysis of the Graz Vitamin D&TT-RCT, a single-center, double-blind, placebo-controlled, parallel-group study performed at the Medical University of Graz, Austria. The trial was designed to investigate the effect of 12 weeks of vitamin D supplementation on TT levels in men.

The methods and study design have been published in detail previously [10,15]. The design, conduction, and publication of this study adhere to the recommendations of the CONSORT Statement (http://www.consort-statement.org/). The trial was registered at http://www.clinicaltrialsregister.eu (EudraCT number, 2011-003575-11) and at clinicaltrials.gov (ClinicalTrials.gov Identifier NCT01748370). The study protocol was approved by the ethics committee of the Medical University of Graz (EK 23-513 ex 10/11) and written informed consent was obtained from each participant before entering the study.

### 2.1. Subjects

Eligible study participants were healthy men aged ≥18 and <70 years with 25(OH)D levels <75 nmol/L. As published previously [10,15], exclusion criteria were hypercalcemia (defined as a serum calcium >2.65 mmol/L), oral or transdermal testosterone supplementation in the last 2 months before entering the study, intramuscular testosterone supplementation 6 months before entering the study, regular intake of vitamin D supplements before study entry, chronic diseases (such as diabetes mellitus), thyroid disease, endocrine disturbances in need of treatment (such as pituitary disorders), history of hypogonadism or known diseases associated with hypogonadism (except obesity) or diseases known to interfere with vitamin D intake or sensitive to vitamin D intake (including inflammatory diseases with granuloma such as sarcoidosis, tuberculosis, Wegener’s granulomatosis, including other forms of vasculitis and inflammatory bowel diseases), intake of medication influencing metabolic or endocrine parameters (insulin sensitizers, insulin, or glucocorticoids) in the last 3 months before study entry, PSA >4 ng/mL (or >3 ng/mL in men at high risk for prostate cancer), palpable prostate nodule or induration, hematocrit >50%, untreated severe obstructive sleep apnea, severe lower urinary tract symptoms, uncontrolled or poorly controlled heart failure, and a history of prostate cancer, breast cancer, orchidectomy, and chromosomal disorders (e.g., Klinefelter syndrome). Men were recruited from the outpatient clinic of the Department of Internal Medicine, Division of Endocrinology and Diabetology, and the outpatient clinic of the Department of Urology, Medical University of Graz, Austria, as well as from male hospital staff and male family members of hospital staff. Men were informed about the trial either by a conversation in the outpatient clinic, by written information posted in the respective outpatient clinics or by a telephone call. All patients were informed that participation in the study is voluntary and that refusal to participate as well as stopping at any time without giving reasons and without any consequences is possible. Written informed consent was obtained before carrying out any study-related procedures from all subjects who participated in the study.

### 2.2. Intervention

Subjects were allocated to the vitamin D or placebo group according to a computer-generated randomization list using a ratio of 1:1. Study medication was placed into numbered bottles according to this computer-generated randomization list. Randomization procedures were conducted using a web-based software (http://www.randomizer.at/) with GCP compliance as confirmed by the Austrian Agency for Health and Food Safety (AGES).

The treatment group received an oral dose of 20,000 IU vitamin D weekly (equivalent to 2857 IU/day) as 50 oily drops weekly (Oleovit D3-drops; Fresenius Kabi Austria GmbH, Linz, Austria) for 12 weeks and the placebo group received 50 oily drops without vitamin D for 12 weeks. Placebo oil contained the same oil as Oleovit D3-drops (without vitamin D content) and was delivered by Fresenius Kabi Austria GmbH, Linz. All investigators who enrolled participants, collected data, and assigned intervention were masked to participant allocation.

To improve and verify compliance, patients were asked to return the study medication bottles (full as well as empty bottles) at the end of study (visit 3).

### 2.3. Outcome Measures

This is a post-hoc analysis of the Graz Vitamin D&TT-RCT investigating vitamin D effects on metabolic parameters (insulin resistance, insulin sensitivity, serum lipids, area under the curve (AUC)glucose, and AUCinsulin), anthropometric parameters (BMI, waist circumference (WC), and waist-to-hip ratio (WHR)), and body composition (fat mass, lean mass, total body fat, fat mass index, and android body fat).

### 2.4. Procedures

Basal blood samples for 25(OH)D, parathyroid hormone (PTH), glucose, insulin, lipids, and calcium were collected between 8.00 and 9.00 a.m. after an overnight fast. Levels of 25(OH)D and TT measured by immunoassays were used for the evaluation of inclusion criteria. Biobanking of remaining blood samples was performed by freezing and storing at −80°C until analysis. Serum levels of 25(OH)D and TT were additionally measured by well-adjusted isotope-dilution liquid chromatography tandem mass spectrometry (ID-LC-MS/MS) methods in 2018 [17,18]. All participants underwent a fasting 75 g oral glucose tolerance test (OGTT). Blood samples were drawn after 30, 60, and 120 min for glucose and insulin determination. AUCglucose and AUCinsulin were calculated according to the trapezoidal method. Insulin resistance was estimated using homeostatic model assessment-insulin resistance (HOMA-IR) and calculated as (fasting plasma insulin (μU/mL) × fasting plasma glucose (mg/dL))/405. QUICKI was used to estimate insulin sensitivity and calculated as 1/log fasting insulin (μU/mL) + log fasting glucose (mg/dL) [19]. To assess β-cell function, HOMA-β was calculated as (20 x fasting insulin (μU/mL))/(fasting glucose (mmol/L) − 3.5). MATSUDA-index was calculated as 10000/√((fasting glucose x fasting insulin)x(mean glucoseOGTT x mean insulinOGTT)) [20].

Body fat and lean mass was measured at baseline and at study end. Fat mass was assessed using DXA scans (iDXA, GE Lunar, Madison, WI, USA) and lean mass was calculated as weight (kg) − fat mass (kg). Two investigators performed all analyses. DXA has been validated previously in children, adults, and the elderly and has been found to be a reliable and valid method for measuring fat mass [17,18]. The coefficient of variation (CV) (i.e., SD from the mean) was evaluated in our laboratory by scanning one person (a female, 30 years of age, 30% body fat, with normal weight and height) seven times in the same day, with repositioning between each scan. For this individual, the CV was 2% for abdominal fat mass and total fat mass. The equipment was calibrated each day using a standardized phantom to detect drifts in measurements, and equipment servicing was performed regularly.

### 2.5. Statistical Analyses

Details on sample size calculation have been published previously [10].

Continuous data are presented as median with interquartile range. The distribution of data was analyzed by descriptive statistics and Kolmogorov–Smirnov test. Skewed variables were log transformed and rechecked for normal distribution. Student’s T-test was used for comparisons of baseline characteristics between groups. Analyses of outcome variables were performed according to the intention-to-treat principle and inclusion of all participants with baseline and follow-up values. Analysis of covariance with adjustments for baseline values was applied to test for differences in the outcome variables between the treatment and the placebo group at study end. We performed subgroup analyses in men with 25(OH)D levels <50 nmol/L. All statistical procedures were performed with SPSS version 23 (SPSS Inc., Chicago, IL, USA). A *p*-value <0.05 was considered statistically significant.

## 3. Results

We took blood samples from ~1100 men and analyzed 25(OH)D and TT concentrations (participant flow charts have been published previously [10,15]). Two-hundred men who gave their written informed consent and met all inclusion as well as no exclusion criteria were randomized and enrolled in the study. The first subject was randomized in December 2012 and the last follow-up was performed in November 2017. We show baseline characteristics of all study participants in Table 1. We found no significant difference in baseline characteristics between the vitamin D and the placebo group. The mean overall treatment period was 86 ± 7 days in the vitamin D and 86 ± 7 days in the placebo group (*p* = 0.422). A total of 192 men completed the study and were analyzed for outcome measures.

We show results of outcome analyses in Table 2. As expected, we found a significant positive vitamin D effect on 25(OH)D levels and a marginally significant trend for a negative effect on PTH levels. Regarding outcome measures, we found a significant negative vitamin D effect on fasting glucose/fasting insulin ratio, whereas we observed no significant effect on the remaining outcome parameters.

In exploratory subgroup analyses among subjects with 25(OH)D levels <50 nmol/L (*n* = 80), we found a significant increase of WC, WHR, total body fat, and android fat in the vitamin D compared to the placebo group (Table 3). Further, we found a trend toward a negative vitamin D effect on QUICKI and HDL-cholesterol levels and increases in fat mass and fat mass index (Table 3).

## 4. Discussion

In this RCT in healthy middle-aged men with 25(OH)D levels <75 nmol/L at baseline, vitamin D treatment had a significant negative effect on fasting glucose/fasting insulin ratio, whereas no significant effect was found on the remaining metabolic parameters or body composition. In men with baseline 25(OH)D levels <50 nmol/L at baseline, we observed a negative vitamin D effect on central obesity and body composition.

Evidence from observational studies has suggested an inverse association of 25(OH)D levels and obesity [8,13,21]. Recently, a cross-sectional analysis including 271 healthy community dwelling seniors found an association of lower 25(OH)D levels with greater fat mass [21]. Further, 25(OH)D was negatively associated with visceral adipose tissue suggesting a link between vitamin D status and fat distribution [13]. Underlying mechanisms for these associations may be a simple sequestration of vitamin D metabolites in the adipose tissue but vitamin D signaling may itself impact on obesity by, e.g., modulation of energy metabolism [22]. Evidence from RCTs on adiposity assessed via gold standard methods (such as DXA or magnetic resonance imaging) is, however, sparse [13,14]. Mousa et al. [14] found no effect on body fat, fat mass, or fat-free mass in 65 overweight or obese subjects receiving either a bolus of 100,000 IU cholecalciferol followed by 4000 IU cholecalciferol or placebo for 16 weeks. Wamberg et al. [13] conducted an RCT among 52 subjects aged 18 to 50 years with BMI >30 kg/m² and 25(OH)D levels <50 nmol/L who were randomized to 26 weeks of treatment with 7000 IU of vitamin D daily or placebo. Vitamin D had no effect on body fat, subcutaneous adipose tissue, or visceral adipose tissue. Similarly, when all men in our trial were analyzed, we found no significant vitamin D effect on body composition including fat mass and fat mass index.

As vitamin D effects might only be seen in subjects with 25(OH)D levels <50 nmol/L, we performed additional analyses in this subgroup. Interestingly, we found a significant adverse effect on surrogate parameters of visceral adiposity (i.e., WC and WHR) as well as on total body fat and android fat. As outlined above, Mousa et al. [14] found no significant effect on parameters of body composition including WHR and body fat assessed by DXA. These differences might be related to study size and participants and different doses (100,000 IU bolus followed by 4000 IU/vitamin D daily versus 20.000 IU/week). One RCT found a positive vitamin D effect on the reduction of fat mass (assessed by Bioelectrical Impedance Analysis) in 77 overweight or obese healthy women receiving 1000 IU vitamin D or placebo daily [23]. However, a recent meta-analysis showed no significant vitamin D effect on weight or BMI [24]. In light of previous results from observational studies on the association of vitamin D and obesity, our findings among men with low vitamin D levels at baseline are unexpected. However, as we found significant vitamin D effects on obesity only in subgroup analyses, our data should be interpreted with caution and the clinical relevance remains to be determined.

We found a significant negative vitamin D effect on fasting glucose/fasting insulin ratio in all men as well as a trend toward decreased QUICKI in men with 25(OH)D levels <50 nmol/L, which is in line with our adverse effects on body composition. Similarly, we observed negative vitamin D effects on QUICKI in healthy men [10] as well as an adverse effect on HOMA-IR and QUICKI in healthy women [25]. However, the majority of RCTs did not support the hypothesis that vitamin D has a significant effect on insulin sensitivity [14,26]. Mousa et al. [14] found no effect of high-dose vitamin D supplementation on insulin sensitivity or insulin secretion assessed by gold-standard methods in 65 overweight or obese subjects. Interestingly, in polycystic ovary syndrome (PCOS) women, some effect on insulin resistance assessed via HOMA-IR has been suggested [27], whereas other RCTs among PCOS women found no significant effect [26]. One study among vitamin D deficient (<50 nmol/L) and insulin resistant women reported a positive effect of 4000 IU cholecalciferol/day over 6 months on insulin resistance and insulin sensitivity [11]. However, our results do not support a favorable role of vitamin D regarding insulin resistance but suggest a potentially harmful effect, which is probably mediated via an unfavorable vitamin D effect on central obesity. From a clinical point of view, there is reason for concern that a growing rate of vitamin D testing and supplementation increases costs and may potentially be harmful. It should be noted that the results of our study do not support the widespread use of vitamin D supplements among healthy subjects. In contrast, until large well-designed RCTs reveal significant beneficial vitamin D effects among healthy subjects without a severe vitamin D deficiency, vitamin D supplementation should only be performed among subjects with really low 25(OH)D levels (<30 nmol/L) [28,29].

When analyzing vitamin D effects on serum lipids, we observed a trend toward an adverse effect of vitamin D treatment on HDL-cholesterol levels. Zittermann et al. [30] reported a positive vitamin D effect on TG levels in 200 healthy overweight subjects receiving 3320 IU vitamin D/day or placebo while participating in a weight-reduction program. In contrast, vitamin D supplementation increased LDL-cholesterol levels. Further, a post-hoc analysis of the Styrian Vitamin D Hypertension Trial suggested a potentially unfavorable effect on lipid metabolism including increases of TG and LDL-cholesterol [12]. A meta-analysis of 12 RCTs detected statistically significant adverse effects on LDL-cholesterol [31], but not on total cholesterol, HDL-cholesterol, and TG. In summary, the effect of vitamin supplementation on serum lipids is currently unclear.

Our study has several limitations that should be mentioned. First, as we investigated men with relatively high 25(OH)D levels at baseline, we cannot exclude significant effects on metabolic parameters in men with lower vitamin D levels. Indeed, our subgroup analyses involving men with low 25(OH)D levels at baseline revealed significant vitamin D effects on body composition. However, as the sample size was relatively small in our subgroup of men with 25(OH)D levels <50 nmol/L, we cannot exclude that using a larger sample size would change our results. Further, despite the design of a RCT, we cannot exclude that confounding factors influenced our results. Our work is a post-hoc analysis and we did not adjust for multiple testing so that our results are prone to statistical type I errors and should be only interpreted in the light of these considerations. Further, our findings are limited to a cohort of healthy middle-aged men and might not be generalizable to other populations such as women or older subjects. The strengths of our study include the design as a RCT, the relatively large sample size as well as the use of a state-of-the-art method for assessing body composition. Our study is the first RCT analyzing vitamin D effects on body composition (assessed via a gold standard method) using vitamin D doses that are close to the doses suggested by current clinical guidelines [32]. Further, vitamin D treatment was effective as reflected by the increase in 25(OH)D levels and the decrease of PTH levels in the vitamin D group.

In summary, we found a possible adverse effect on insulin sensitivity as mirrored by a significant vitamin D effect on fasting glucose/fasting insulin ratio. To the best of our knowledge, we are the first demonstrating that in vitamin D deficient men, vitamin supplementation had an adverse effect on obesity as reflected by increases in surrogate parameters of visceral adiposity (i.e., WC and WHR), body fat, and android fat. In light of these and previous data, it is unlikely that vitamin D supplementation improves obesity or cardiovascular risk factors but might even have a potential harmful effect on obesity and insulin sensitivity. When considering the wide use of vitamin D testing and treatment, further RCTs and meta-analyses of RCTs adequately powered to address the potential harmful effects of vitamin D on obesity, and cardiovascular risk factors are of high clinical importance.

## Figures and Tables

**Table 1:** Baseline characteristics of study participants. Comparisons of baseline characteristics between men in the vitamin D and the placebo group were performed using student’s T-test.

	All Study Participants (*n* = 192)		Vitamin D (*n* = 96)		Placebo (*n* = 96)		
	Median	IQR	Median	IQR	Median	IQR	*p*-*Value*
**Age (Years)**	45	31–54	40	30–53	47	32–55	0.230
**25-Hydroxyvitamin D (nmol/L)**	53	42–68	53	43–68	52	42–64	0.847
**Parathyroid Hormone (pg/mL)**	45.8	35.8–55.6	46.6	36.2–57.0	43.4	35.8–54.3	0.611
**HOMA-IR**	2.2	1.3–3.7	2.2	1.2–3.6	2.3	1.5–3.9	0.274
**HOMA-β**	136.5	90.2–211.3	130.1	85.1–190.5	150.8	98.2–217.3	0.238
**MATSUDA-Index**	7.3	0.4–10.3	7.3	0.3–10.7	7.9	4.1–10.3	0.938
**QUICKI**	0.34	0.32–0.37	0.34	0.32–0.37	0.34	0.31–0.36	0.323
**AUCglucose**	231.1	196.8–272.3	231.9	197.9–273.3	229.9	193.8–262.0	0.375
**AUCinsulin**	100.6	61.0–159.7	104.0	64.8–148.8	98.6	59.5–162.5	0.296
**Fasting Glucose/Fasting Insulin-Ratio**	9.0	6.1–14.0	9.6	6.3–16.0	8.5	6.0–12.4	0.429
**Proinsulin (mU/L)**	7.8	6.4-9.9	7.8	6.5–10.5	7.6	6.0–9.4	0.230
**Total Cholesterol (mg/dL)**	200	171–224	190	164–222	208	180–224	0.150
**HDL-C (mg/dL)**	55	44–65	56	45–65	54	44–65	0.815
**LDL-C (mg/dL)**	117	93–142	110	89–139	122	98–144	0.258
**Triglycerides (mg/dL)**	99	69–151	99	67–133	100	75–170	0.275
**Body Mass Index (kg/m²)**	26.4	24.1–29.5	26.3	24.0–29.3	26.8	24.1–29.8	0.548
**Waist Circumference (cm)**	93	86-102	90	86–100	94	87–104	0.244
**Waist-to-Hip Ratio**	0.92	0.87-0.96	0.91	0.86–0.95	0.93	0.88–0.97	0.248
**Fat Mass (kg)**	23.9	18.3-31.1	23.1	18.0–29.9	24.9	18.8–32.6	0.396
**Lean Mass (kg)**	58.6	55.1–63.6	58.5	54.9–64.3	58.7	55.2–63.5	0.777
**Total Body Fat (%)**	29.2	23.6–34.0	28.0	23.1–32.8	29.9	23.8–35.0	0.396
**Android Fat (%)**	38	28–45	35	26–44	39	27–44	0.395
**Fat Mass Index (%)**	13.3	10.3–17.6	13.0	10.1–16.6	13.7	10.6–18.6	0.423

HOMA-IR, homeostasic model assessment-insulin resistance; IQR, interquartile range; QUICKI, quantitative insulin sensitivity check index; AUC, area under the curve; HDL-C, high density lipoprotein-cholesterol; LDL-C, low density lipoprotein-cholesterol.

**Table 2:** Continuous outcome variables at baseline and follow-up at study end (12 weeks) in study participants with available values at both study visits. Data are shown as medians and interquartile range. Treatment effects with 95% confidence interval and *p*-values were calculated by ANCOVA for group differences at follow-up with adjustment for baseline values.

	Baseline Visit		Study End		Treatment Effect		
	Median	IQR	Median	IQR	Between Group Differences with 95 % CI		*p-Value*
***Endocrine Characteristics***							
**25-Hydroxyvitamin D** (**nmol/L)**							
Vitamin D (*n* = 96)	53	43–68	98	85–116	37	31 to 44	<0.001
Placebo (*n* = 96)	52	42–64	65	51–77			
**Parathyroid hormone (pg/mL)**							
Vitamin D (*n* = 96)	46.6	36.2–57.0	46.4	35.0–59.2	−0.09	−0.17 to −0.04	0.021
Placebo (*n* = 96)	43.4	35.8–54.3	49.3	38.7–61.6			
***Metabolic Characteristics***							
**Homeostasic Model Assessment-Insulin Resistance**							
Vitamin D (n = 38)	2.2	1.2–3.6	2.3	1.5–3.4	0.171	−0.032 to 0.373	0.098
Placebo (n = 42)	2.3	1.5-3.9	2.2	1.4–3.7			
**Homeostasic Model Assessment-β**							
Vitamin D (n = 38)	130.1	85.1–190.5	142.0	102.6–206.5	0.145	−0.043 to 0.332	0.131
Placebo (n = 42)	150.8	98.2–217.3	138.3	94.5–209.6			
**MATSUDA-Index**							
Vitamin D (*n* = 38)	7.3	0.3–10.7	5.3	3.5–8.9	0.091	−0.094 to .276	0.332
Placebo (*n* = 42)	7.9	4.1–10.3	5.2	3.2–8.3			
**Quantitative Insulin Sensitivity Check Index**							
Vitamin D (*n* = 92)	0.34	0.32–0.37	0.34	0.32–0.36	−0.03	−0.62 to 0.03	0.075
Placebo (*n* = 92)	0.34	0.31–0.36	0.34	0.31–0.36			
**Area Under the Curve Glucose**							
Vitamin D (*n* = 38)	231.9	197.9–273.3	229.1	200.3–269.8	0.001	−0.45 to 0.048	0.954
Placebo (*n* = 42)	229.9	193.8–262.0	226.9	199.4–272.5			
**Area Under the Curve Insulin**							
Vitamin D (*n* = 38)	104.0	64.8–148.8	94.9	54.7–150.7	−0.056	−0.270 to 0.158	0.605
Placebo (*n* = 42)	98.6	59.5–162.5	95.9	61.2–153.4			
**Fasting Glucose/Fasting Insulin-Ratio**							
Vitamin D (*n* = 92)	9.6	6.3–16.0	8.9	6.2–12.4	−5.30	−10.4 to −0.2	0.040
Placebo (*n* = 92)	8.5	6.0–12.4	9.4	5.7–13.2			
**Proinsulin (mU/L)**							
Vitamin D (*n* = 74)	7.8	6.5–10.5	8.1	6.2–10.7	0.039	−0.004 to 0.082	0.077
Placebo (*n* = 75)	7.6	6.0–9.4	7.4	6.1–9.4			
***Lipids***							
**Total Cholesterol (mg/dL)**							
Vitamin D (*n* = 85)	190	164–222	191	166–225	1.6	−5.2 to 8.4	0.641
Placebo (*n* = 84)	208	180–224	199	171–230			
**High Density Lipoprotein-Cholesterol (mg/dL)**							
Vitamin D (*n* = 84)	56	45–65	55	43–68	−0.8	−3.4 to 1.9	0.581
Placebo (*n* = 83)	54	44–65	56	47–67			
**Low Density Lipoprotein-Cholesterol (mg/dL)**							
Vitamin D (*n* = 82)	110	89–139	117	90–142	2.3	−4.0 to 8.5	0.477
Placebo (*n* = 79)	122	98–144	117	96–143			
**Triglycerides (mg/dL)**							
Vitamin D (*n* = 85)	99	67–133	96	73–146	−0.007	−0.121 to 0.107	0.898
Placebo (*n* = 84)	100	75–170	105	73–160			
***Body Composition***							
**Body Mass Index (kg/m²)**							
Vitamin D (*n* = 96)	26.3	24.0–29.3	26.3	24.2–28.9	0.004	−0.004 to 0.012	0.274
Placebo (*n* = 96)	26.8	24.1–29.8	26.6	24.0–29.5			
**Waist Circumference (cm)**							
Vitamin D (*n* = 96)	90	86–100	93	85–102	0.02	−0.07 to 0.012	0.609
Placebo (*n*= 96)	94	87–104	94	87–102			
**Waist-to-Hip Ratio**							
Vitamin D (*n* = 96)	0.91	0.86–0.95	0.92	0.87–0.96	0.00	−0.10 to 0.10	0.969
Placebo (*n* = 96)	0.93	0.88–0.97	0.93	0.87–0.98			
**Fat Mass (kg)**							
Vitamin D (*n* = 96)	23.1	18.0–29.9	23.0	18.2–30.0	0.009	−0.012 to 0.030	0.388
Placebo (*n* = 95)	24.9	18.8–32.6	24.8	18.9–31.2			
**Lean Mass (kg)**							
Vitamin D (*n* = 96)	58.5	54.9–64.3	58.3	54.1–64.0	−0.017	−0.38 to 0.34	0.927
Placebo (*n* = 95)	58.7	55.2–63.5	58.7	55.4–63.2			
**Total Body Fat (%)**							
Vitamin D (*n* = 96)	28.0	23.1–32.8	28.6	24.1–32.6	0.010	−0.008 to 0.029	0.270
Placebo (*n* = 95)	29.9	23.8–35.0	29.8	24.4–34.4			
**Android Fat (%)**							
Vitamin D (*n* = 96)	35	26–44	36	27–44	0.020	−0.010 to 0.050	0.184
Placebo (*n* = 95)	39	27–44	39	29–44			
**Fat mass Index**							
Vitamin D (*n* = 96)	13.0	10.1–16.6	12.9	10.3–17.1	0.009	−0.012 to 0.030	0.390
Placebo (*n* = 95)	13.7	10.6–18.6	13.7	10.6–17.3			

Subgroup analyses: subjects with 25(OH)D levels <50 nmol/L.

**Table 3:** Continuous outcome variables at baseline and follow-up at study end (12 weeks) in study participants with 25 hydroxyvitamin D <50 nmol/L at baseline and available values at both study visits. Data are shown as medians and interquartile range. Treatment effects with 95% confidence interval and *p*-values were calculated by ANCOVA for group differences at follow-up with adjustment for baseline values.

	Baseline Visit		Study End		Treatment Effect		
	Median	IQR	Median	IQR	Between Group Differences with 95 % CI		*p*-Value
***Endocrine Characteristics***							
**25-Hydroxyvitamin D** (**nmol/L)**							
Vitamin D (*n* = 38)	40	34–44	89	79–107	44	35 to 53	<0.001
Placebo (*n* = 42)	42	31–46	53	37–64			
**Parathyroid Hormone (pg/mL)**							
Vitamin D (*n* = 38)	50.1	44.8–58.7	47.0	33.9–63.9	−0.12	−0.24 to 0.00	0.058
Placebo (*n* = 42)	47.5	40.3–56.9	49.0	40.3–58.2			
***Metabolic characteristics***							
**Homeostasic Model Assessment-Insulin Resistance**							
Vitamin D (*n* = 38)	2.0	1.1–3.6	2.5	1.3–3.1	−0.3	−1.0 to 0.9	0.955
Placebo (*n* = 42)	2.1	1.5–3.4	1.9	1.1–3.2			
**Homeostasic Model Assessment-β**							
Vitamin D (*n* = 38)	124.7	82.4–217.0	142.0	101.6–209.4	−0.6	−54.0 to 52.7	0.982
Placebo (*n* = 42)	164.2	112.2–221.5	130.9	93.3–196.0			
**MATSUDA-Index**							
Vitamin D (*n* = 38)	8.5	0.4–10.9	6.0	3.4–8.9	0.3	−1.1 to 1.7	0.647
Placebo (*n* = 42)	8.4	4.8–10.1	5.8	3.3–8.5			
**Quantitative Insulin Sensitivity Check Index**							
Vitamin D (*n* = 38)	0.34	0.32–0.38	0.33	0.32–0.37	−0.01	−0.25 to 0.01	0.061
Placebo (*n* = 42)	0.34	0.32–0.36	0.35	0.32–0.38			
**Area Under the Curve Glucose**							
Vitamin D (*n* = 38)	227.5	213.0–273.8	233.1	202.5–275.8	−8.4	−28.5 to 11.6	0.404
Placebo (*n* = 42)	222.5	181.8–261.5	220.0	190.5–265.8			
**Area Under the Curve Insulin**							
Vitamin D (*n* = 38)	90.5	68.2–128.5	103.5	61.5–172.2	−12.7	−69.6 to 44.1	0.657
Placebo (*n* = 42)	84.2	63.9–128.1	91.7	63.9–128.1			
**Fasting Glucose/Fasting Insulin Ratio**							
Vitamin D (*n* = 38)	10.0	6.0–16.7	9.3	6.1–12.3	−10.1	−21.6 to 1.4	0.085
Placebo (*n* = 42)	8.6	6.1–12.4	9.9	6.4–14.3			
**Proinsulin (mU/L)**							
Vitamin D (*n* = 38)	8.8	7.0–11.7	8.6	5.9–11.3	0.056	−0.018 to 0.130	0.136
Placebo (*n* = 42)	8.4	6.8–9.7	8.2	6.5–10.2			
***Lipids***							
**Total Cholesterol (mg/dL)**							
Vitamin D (*n* = 33)	194	166–230	199	177–224	2.7	−7.4 to 12.8	0.592
Placebo (*n* = 37)	208	171–219	196	165–218			
**High Density Lipoprotein-Cholesterol (mg/dL)**							
Vitamin D (*n* = 33)	55	42–63	48	41–64	−3.8	−7.8 to 0.3	0.070
Placebo (*n* = 37)	55	44–65	56	47–64			
**Low Density Lipoprotein-Cholesterol (mg/dL)**							
Vitamin D (*n* = 33)	111	89–139	123	91–144	5.3	3.8 to 14.1	0.238
Placebo (*n* = 37)	120	93–141	113	96–136			
**Triglycerides (mg/dL)**							
Vitamin D (*n* = 36)	122	78–161	109	78–160	0.02	−0.17 to 0.21	0.821
Placebo (*n* = 39)	100	63–162	104	64–159			
***Body Composition***							
**Body Mass Index (kg/m²)**							
Vitamin D (*n* = 38)	26.8	24.9–31.4	27.3	25.3–31.3	0.23	−0.14 to 0.60	0.161
Placebo (*n* = 42)	26.4	23.5–29.4	26.3	23.6–29.3			
**Waist Circumference (cm)**							
Vitamin D (*n* = 38)	94	86–103	94	84–104	1.6	0.3 to 2.9	0.012
Placebo (*n* = 42)	93	84–104	92	83–99			
**Waist-to-Hip Ratio**							
Vitamin D (*n* = 38)	0.91	0.85–0.95	0.93	0.87–0.97	0.019	0.002 to 0.036	0.031
Placebo (*n* = 42)	0.91	0.87–0.97	0.91	0.85–0.97			
**Fat Mass (kg)**							
Vitamin D (*n* = 38)	25.8	18.3–33.4	26.0	18.4–35.0	0.031	−0.001 to 0.063	0.058
Placebo (*n* = 42)	24.0	14.5–30.8	23.6	14.9–28.9			
**Lean Mass (kg)**							
Vitamin D (*n* = 38)	61.0	55.3–66.2	60.2	54.2–66.4	−0.067	−0.64 to 0.51	0.818
Placebo (*n* = 42)	58.5	55.7–61.6	58.5	56.4–61.8			
**Total Body Fat (%)**							
Vitamin D (*n* = 38)	29.5	24.8–34.9	30.1	24.6–36.4	0.029	0.004 to 0.055	0.026
Placebo (*n* = 42)	28.6	20.6–34.4	27.9	20.9–33.6			
**Android Fat (%)**							
Vitamin D (*n* = 38)	39	25–46	38	22–45	1.18	0.11 to 2.26	0.010
Placebo (*n* = 42)	38	28–47	36	22–44			
**Fat Mass Index**							
Vitamin D (*n* = 38)	13.9	10.2–18.7	14.1	10.8–19.0	0.031	−0.001 to 0.063	0.057
Placebo (*n* = 42)	13.1	8.4–17.3	13.0	8.5–16.7			
